# Supplementary material for: Untangling the systematic dilemma behind the roughskin spurdog Cirrhigaleus asper (Merrett, 1973) (Chondrichthyes: Squaliformes), with phylogeny of Squalidae and a key to Cirrhigaleus species
Source: PLoS One. 2023 Mar 6;18(3):e0282597. doi: 10.1371/journal.pone.0282597 (PMC9987817; doi:10.1371/journal.pone.0282597)
Supplement: S1 Table — 1, monospondylous vertebral counts; 2, diplospondylous vertebral counts; 3, upper tooth row counts; 4, lower tooth row counts. Absolute values and normalized ones (in parentheses) are given. (DOCX) [file pone.0282597.s001.docx]

**Supporting information**

Viana and Soares. 2022. Untangling the systematic dilemma behind the roughskin spurdog *Cirrhigaleus asper* (Merrett, 1973) (Chondrichthyes: Squaliformes), with phylogeny of Squalidae and a key to *Cirrhigaleus* species.

**Table S1. Matrix summarizing quantitative characters used in the phylogenetic study.** 1, monospondylous vertebral counts; 2, diplospondylous vertebral counts; 3, upper tooth row counts; 4, lower tooth row counts. Absolute values and normalized ones (in parentheses) are given.

| **Terminal** | **1** | **2** | **3** | **4** |
| --- | --- | --- | --- | --- |
| *Dalatias licha* | 40  (0.188) | 43  (0.190) | 16–21  (0.000-0.217) | 17–20  (0.125-0.313) |
| *Isistius brasiliensis* | 37–44  (0.000-0.438) | 35–47  (0.00-0.286) | 17–39  (0.043-1.000) | 15–31  (0.000-1.000) |
| *Squalus acanthias* | 46–50  (0.563-0.813) | 60–68  (0.595-0.786) | 25–30  (0.391-0.609) | 21–23  (0.375-0.500) |
| *Squalus suckleyi* | 37–43  (0.000-0.375) | 61–69  (0.619-0.810) | 24–29  (0.348-0.565) | 18–24  (0.188-0.563) |
| *Squalus megalops* | 37–41  (0.000-0.250) | 64–69  (0.690-0.810) | 23–28  (0.304-0.522) | 22–27  (0.438-0.750) |
| *Squalus brevirostris* | 39–42  (0.125-0.313) | 63–68  (0.667-0.786) | 26–28  (0.435-0.522) | 22–24  (0.438-0.563) |
| *Squalus albifrons* | 43–47  (0.375-0.625) | 60–76  (0.595-0.976) | 27–28  (0.478-0.522) | 22–24  (0.438-0.563) |
| *Squalus mitsukurii* | 42–46  (0.313-0.563) | 63–72  (0.667-0.881) | 21–27  (0.217-0.478) | 17–24  (0.125-0.563) |
| *Squalus montalbani* | 38–47  (0.063-0.625) | 66–72  (0.738-0.881) | 22–26  (0.261-0.435) | 18–24  (0.188-0.563) |
| *Squalus japonicus* | 41–43  (0.250-0.375) | 72–77  (0.881-1.00) | 23–27  (0.304-0.478) | 18–24  (0.188-0.563) |
| *Cirrhigaleus barbifer* | 47–50  (0.625-0.813) | 68–72  (0.786-0.881) | 22–28  (0.261-0.522) | 20–25  (0.313-0.625) |
| *Cirrhigaleus australis* | 49–53  (0.750-1.00) | 73–74  (0.905-0.929) | 25–27  (0.391-0.478) | 20–23  (0.313-0.500) |
| *Cirrhigaleus asper* | 49–52  (0.750-0.938) | 65–75  (0.714-0.952) | 24–27  (0.348-0.478) | 20–24  (0.313-0.563) |
