# Supplementary material for: Untangling the systematic dilemma behind the roughskin spurdog Cirrhigaleus asper (Merrett, 1973) (Chondrichthyes: Squaliformes), with phylogeny of Squalidae and a key to Cirrhigaleus species
Source: PLoS One. 2023 Mar 6;18(3):e0282597. doi: 10.1371/journal.pone.0282597 (PMC9987817; doi:10.1371/journal.pone.0282597)
Supplement: S2 Table — (DOCX) [file pone.0282597.s002.docx]

**Supporting Information**

Viana and Soares. 2022. Untangling the systematic dilemma behind the roughskin spurdog *Cirrhigaleus asper* (Merrett, 1973) (Chondrichthyes: Squaliformes), with phylogeny of Squalidae and a key to *Cirrhigaleus* species.

**S2 Table. Matrix of discrete morphological characters utilized in the phylogenetic analysis.**

*Dalatias licha*

00000?0?0?0000??00000000???00001?0?00000000?0?0

*Isistius brasiliensis*

00000?0?0?0000??0000100000000001?0?00000110?0?0

*Squalus acanthias*

0011111010011110100001121011111[01]?11100011110112

*Squalus suckleyi*

0011111010011110100001121011111[01]?11100011110112

*Squalus megalops*

00111110100111110000010000111110?11111111110103

*Squalus brevirostris*

00111110100111110000010000111110?11111111110103

*Squalus albifrons*

00111110100111110010110000111110?11111111110103

*Squalus mitsukurii*

00111111110111110010011100111110?11111111110101

*Squalus montalbani*

00111111112111110011111100111110?11111111110101

*Squalus japonicus*

00111111111111110010111000111110?11111111110101

*Cirrhigaleus barbifer*

1101100?112011010111121101?11100110101001111101

*Cirrhigaleus australis*

1101100?112011010111121101?11100110101001111101

*Cirrhigaleus asper*

1101100?112011010111121101?11100010101001110101
