# Supplementary material for: Untangling the systematic dilemma behind the roughskin spurdog Cirrhigaleus asper (Merrett, 1973) (Chondrichthyes: Squaliformes), with phylogeny of Squalidae and a key to Cirrhigaleus species
Source: PLoS One. 2023 Mar 6;18(3):e0282597. doi: 10.1371/journal.pone.0282597 (PMC9987817; doi:10.1371/journal.pone.0282597)
Supplement: S1 File — (DOCX) [file pone.0282597.s005.docx]

**Supporting information**

Viana and Soares. 2022. Untangling the systematic dilemma behind the roughskin spurdog *Cirrhigaleus asper* (Merrett, 1973) (Chondrichthyes: Squaliformes), with phylogeny of Squalidae and a key to *Cirrhigaleus* species.

**S1 File. List of** **non-ambiguous synapomorphies of clades and terminal taxa based on the two equally most-parsimonious cladograms resulting from implied weighting (*k* = 1).**

Clade A

Char. 2: 0.190-0.286>0.690-0.786

Char. 8: 0 > 1

Char. 9: 0 > 1

Char. 13: 0 > 1

Char. 17: 0 > 1

Char. 18: 0 > 1

Char. 26: 0 > 1

Char. 27: 0 > 1

Char. 28: 1 > 0

Char. 32: 1 > 0

Char. 33: 0 > 1

Char. 34: 0 > 1

Char. 36: 0 > 1

Char. 40: 1 > 0

Char. 42: 0 > 1

Char. 47: 0 > 1

Char. 49: 0 > 1

Char. 51: 0 > 1

Clade B

Char. 7: 0 >1

Char. 11: 0 > 1

Char. 16: 0 > 1

Char. 35: 0 > 1

Char. 44: 0 > 1

Clade C

Char. 20: 1 > 0

Char. 21: 0 > 1

Char. 28: 1 > 2

Char. 29: 0 > 1

Char. 50: 0 > 1

Char. 51: 1 > 2

*Squalus acanthias*:

Char. 1: 0.375>0.563-0.813

*Squalus suckleyi*:

No autapomorphies

Clade D

Char. 2: 0.690-0.786>0.881

Char. 41: 0 > 1

Char. 43: 0 > 1

Clade E

Char. 27: 1 > 0

Char. 28: 1 > 0
Char. 51: 1 > 3

*Squalus albifrons*:

No autapomorphies

Clade F

Char. 1: 0.375>0.250-0.313

*Squalus megalops*:

No autapomorphies

*Squalus brevirostris*:

No autapomorphies

Clade G

Char. 12: 0 > 1

*Squalus mitsukurii*:

No autapomorphies

Clade H

Char. 15: 0 > 1

*Squalus japonicus*:

No autapomorphies

*Squalus montalbani*:

Char. 14: 01 > 2

Char. 24: 0 > 1

Clade I

Char. 1: 0.375>0.750

Char. 5: 0 > 1

Char. 6: 0 > 1

Char. 15: 1 > 2

Char. 22: 0 > 1

Char. 24: 0 > 1

Char. 30: 1 > 2

*Cirrhigaleus asper*:

No autapomorphies

Clade J

Char. 48: 0 > 1

*Cirrhigaleus barbifer*:

No autapomorphies

*Cirrhigaleus australis*:

No autapomorphies
