# Supplementary material for: Untangling the systematic dilemma behind the roughskin spurdog Cirrhigaleus asper (Merrett, 1973) (Chondrichthyes: Squaliformes), with phylogeny of Squalidae and a key to Cirrhigaleus species
Source: PLoS One. 2023 Mar 6;18(3):e0282597. doi: 10.1371/journal.pone.0282597 (PMC9987817; doi:10.1371/journal.pone.0282597)
Supplement: S2 File — (DOCX) [file pone.0282597.s006.docx]

**Supporting information**

Viana and Soares. 2022. Untangling the systematic dilemma behind the roughskin spurdog *Cirrhigaleus asper* (Merrett, 1973) (Chondrichthyes: Squaliformes), with phylogeny of Squalidae and a key to *Cirrhigaleus* species.

**S2 File. List of morphological character transformation based on the two equally most-parsimonious cladograms resulting from implied weighting (k = 1).**

Char. 1 (L = 875):

Clade A: 0.188-0.375>0.375

*Squalus japonicus*: 0.375> 0.250-0.375

*Squalus montalbani*: 0.37>0.063-0.625

*Squalus suckleyi*: 0.375>0.00-0.375

*Squalus acanthias*: 0.37> 0.563-0.813

*Squalus mitsukurii*: 0.375>0.313-0.563

*Squalus albifrons*: 0.375>0.375-0.625

Clade G: 0.375>0.250-0.313

*Squalus brevirostris*: 0.25-0.31>0.12-0.31

*Squalus megalops*: 0.250-0.313>0.00-0.25

Clade I: 0.375>0.750

*Cirrhigaleus_asper*: 0.750>0.750-0.938

*Cirrhigaleus_australis*: 0.750>0.750-1.00

*Cirrhigaleus_barbifer*: 0.750>0.625-0.813

Char. 2 (L= 810):

Clade A 19: 0.190-0.286>0.69-0.786

Clade B: 0.690-0.786>0.786-0.881

*Squalus japonicus*: 0.78-0.88>0.714-0.952

*Squalus montalbani*: 0.78-0.88>0.90-0.93

*Squalus albifrons*: 0.786-0.88>0.88-1.00

*Squalus suckleyi*: 0.786-0.88>0.595-0.976

*Squalus acanthias*: 0.786-0.88>0.667-0.78

*Squalus brevirostris*: 0.786-0.88>0.73-0.88

*Squalus megalops*: 0.78-0.88>0.66-0.88

*Cirrhigaleus asper*: 0.69-0.786>0.69-0.81

*Cirrhigaleus australis*: 0.69-0.79>0.62-0.8

*Cirrhigaleus barbifer*: 0.69-0.78>0.59-0.78

Char. 3 (L= 261):

Clade A: 0.22-0.43>0.391-0.435

*Squalus japonicus*: 0.39-0.435>0.35-0.478

*Squalus montalbani*: 0.39-0.43>0.39-0.48

*Squalus mitsukurii*: 0.39-0.435>0.26-0.522

Clade E: 0.391-0.435>0.435-0.48

*Squalus albifrons*: 0.391-0.435>0.30-0.48

*Squalus suckleyi*: 0.435-0.48>0.48-0.522

*Squalus acanthias*: 0.435-0.48>0.435-0.52

*Squalus brevirostris*: 0.391-0.43>0.26-0.43

*Squalus megalops*: 0.39-0.435>0.217-0.48

*Cirrhigaleus asper*: 0.39-0.435>0.30-0.52

*Cirrhigaleus australis*: 0.4-0.43>0.35-0.56

*Cirrhigaleus barbifer*: 0.39-0.43>0.39-0.61

Char. 4 (L= 125):

Clade A: 0.31-0.44>0.44

*Squalus japonicus*: 0.44>0.31-0.56

*Squalus montalbani*: 0.44>0.31-0.50

*Squalus mitsukurii*: 0.44>0.313-0.625

*Squalus albifrons*: 0.44>0.19-0.56

*Squalus suckleyi*: 0.44>0.438-0.563

*Squalus acanthias*: 0.44> 0.438-0.563

*Squalus brevirostris*: 0.44>0.188-0.563

*Squalus megalops*: 0.44>0.125-0.563

*Cirrhigaleus asper*: 0.44>0.44-0.750

*Cirrhigaleus australis*: 0.44>0.188-0.56

*Cirrhigaleus barbifer*: 0.44>0.375-0.50

Char. 5 (L= 1):

Clade I: 0 > 1

Char. 6 (L= 1):

Clade I: 0 > 1

Char. 7 (L= 2):

Clade B: 01 > 1

*Cirrhigaleus asper*: 0 > 01

Char. 8 (L= 1):

Clade A: 0 > 1

Char. 9 (L= 1):

Clade A: 0 > 1

Char. 10 (L= 1):

Clade B: 01 > 1

Clade I: 01 > 0

Char. 11 (L= 1):

Clade B: 0 > 1

Char. 12 (L= 1):

Clade G: 1 > 0

Char. 13 (L= 1):

Clade A: 0 > 1

Char. 14 (L= 1):

Clade E: 1 > 0

Char. 15 (L= 4):

Clade I: 0 > 2

Clade H: 0 > 1

*Squalus montalbani*: 1 > 2

Char. 16 (L= 1):

Clade B: 0 > 1

Char. 17 (L= 1):

Clade A: 0 > 1

Ch. 18 (L= 1):

Clade A: 0 > 1

Char. 19 (L= 2):

Clade B: 01 > 0

Clade I: 01 > 1

Char. 20 (L= 1):

Clade C: 1 > 0

Char. 21 (L= 1):

Clade C: 0 > 1

Char. 22 (L= 1):

Clade B: 0 > 1

Char. 23 (L= 5):

Clade A: 01 > 1

Clade C: 01 > 0

Clade G: 01 > 1

Clade H: 01 > 0

*Squalus albifrons*: 01 > 1

Char. 24 (L= 3):

*Squalus montalbani*: 0 > 1

Clade A: 0 > 1

Char. 25 (L= 3):

Clade A: 01 > 0

Clade C: 01 > 0

Clade H: 01 > 1

*Squalus albifrons*: 0 > 1

*Squalus mitsukurii*: 01 > 1

Clade F: 01 > 0

Char. 26 (L= 2):

Clade A: 0 > 1

Clade I: 1 > 2

Char. 27 (L= 2):

Clade A: 0 > 1

Clade E: 1 > 0

Char. 28 (L= 3):

Clade A: 0 > 1

Clade C: 1 > 2

Clade E: 1 > 0

*Squalus japonicus*: 1 > 0

Char. 29 (L= 1):

Clade C: 0 > 1

Char. 30 (L= 1):

Clade I: 0 > 1

Char. 31 (L= 1):

Clade B: 01 > 1

Char. 32 (L= 1):

Clade A: 0 > 1

Char. 33 (L= 1):

Clade A4 0 > 1

Char. 34 (L= 1):

Clade A: 0 > 1

Char. 35 (L= 1):

Clade B: 0 > 1

Char. 36 (L= 1):

Clade A: 1 > 0

*Squalus suckleyi*: 0 > 01

*Squalus acanthias*: 0 > 01

Char. 37 (L= 1):

Clade J: 01 > 1

*Cirrhigaleus asper*: 01 > 0

Char. 38 (L= 1):

Clade A: 0 > 1

Char. 39 (L= 2):

Clade B: 01 > 1

Clade H: 01 > 0

Char. 40 (L= 1):

Clade A: 0 > 1

Char. 41 (L= 1):

Clade D: 0 > 1

Char. 42 (L= 2):

Clade A: 0 > 01

Clade I: 01 > 0

Char. 43 (L= 1):

Clade D: 0 > 1

Char. 44 (L= 1):

Clade B: 0 > 1

Char. 45 (L= 1):

No transformation in Squalidae.

Char. 46 (L= 1):

No transformation in Squalidae.

Char. 47 (L= 1):

Clade A: 0 > 1

Char. 48 (L= 1):

Clade J: 0 > 1

Char. 49 (L=):

Clade A: 0 > 1

Char. 50 (L= 1):

Clade C: 0 > 1

Char. 51 (L= 3):

Clade A: 0 > 1

Clade C: 1 > 2

Clade E: 1 > 3
