# Supplementary material for: Untangling the systematic dilemma behind the roughskin spurdog Cirrhigaleus asper (Merrett, 1973) (Chondrichthyes: Squaliformes), with phylogeny of Squalidae and a key to Cirrhigaleus species
Source: PLoS One. 2023 Mar 6;18(3):e0282597. doi: 10.1371/journal.pone.0282597 (PMC9987817; doi:10.1371/journal.pone.0282597)
Supplement: S5 File — Preserved examined material of Cirrhigaleus species are given in full under the section ‘Taxonomic account’. Skeletal material for character analysis and polarity is given for both ingroups and outgroups of the current analysis. (DOCX) [file pone.0282597.s009.docx]

**Supporting information**

Viana and Soares. 2022. Untangling the systematic dilemma behind the roughskin spurdog *Cirrhigaleus* *asper* (Merrett, 1973) (Chondrichthyes: Squaliformes), with phylogeny of Squalidae and a key to *Cirrhigaleus* species.

**S5 File. A list of examined preserved material is provided below and include species of the genera *Squalus, Dalatias* and *Isistius* that were used for comparative purposes.** Preserved examined material of *Cirrhigaleus* species are given in full under the section ‘Taxonomic account’. Skeletal material for character analysis and polarity is given for both ingroups and outgroups of the current analysis.

Preserved material: *S. acanthias*: NRM 21757, adult male, 690 mm TL, Sweden; NRM 21758, adult female, 715 mm TL, Sweden; NRM 21759, adult female, 775 mm TL, Sweden; NRM 21762, juvenile female, 420mm TL, Sweden; NRM 21763, adult male, 645 mm TL, Sweden; NRM 21767, adult male, 630 mm TL, Sweden; NRM 44740, adult female, 830 mm TL, Sweden; and material listed in Viana and Carvalho (2020). *S. suckleyi*: CAS 21971, two juvenile females, 310–315 mm TL, three juvenile males, 310–330 mm TL, California, USA; CAS 227267 (neotype), adult male, 674 mm TL, Washington State, USA; CAS 227269, adult male, 691 mm TL, Washington State, USA; CAS 227270, adult male, 730 mm TL, Washington State, USA; CAS 227271, adult male, 800 mm TL, Washington State, USA; CAS 227272, adult male, 640 mm TL, Washington State, USA; CAS 227273, adult male, 700 mm TL, Washington State, USA; and material listed in Viana and Carvalho (2020). *S. megalops*: AMS I 16255-001 (holotype), adult female, 550 mm TL, Australia; CSIRO H 6482-02, adult male, 426 mm TL, Australia. *S. brevirostris, S. japonicus* and *S. mitsukurii*: material listed in Viana and Carvalho (2020). *S. montalbani*: CSIRO H 2609-07, adult male, 620 mm TL, Western Australia; USNM 70256 (holotype), juvenile male, 311 mm TL, off Sombrero Island, Philippines. *S. albifrons*: CSIRO H 4627-01 (holotype), adult male, 615 mm TL, Australia; CSIRO CA 3297 (paratype of *S. altipinnis*), adult male, 585 mm TL, Western Australia. *D. licha*: CAS 224577, juvenile male, 367mm TL, Taiwan; SAIAB 6057, juvenile male, 404 mm TL, South Africa; SAIAB 6058, juvenile female, 406 mm TL, Mozambique; SAIAB 6059, juvenile male, 398 mm TL, South Africa; SAIAB 6236, adult male, 1123mm TL, South Africa; SAIAB 13019, adult female, unknown TL, no data; SAIAB 13020, adult female, 884mm TL, unknown TL, no data; SAIAB 81691, juvenile male, 402 mm TL, Mozambique; SAIAB 189052, juvenile female, 428 mm TL, South Africa; SAIAB 193025, juvenile male, 395 mm TL, Mozambique. *I. brasiliensis*: SAIAB 64998, juvenile female, 403 mm TL, Angola; SAIAB 65007, female 400 mm TL, Angola; SAIAB 65972, female 300 mm TL, Angola.

Skeletal material. *C. barbifer* (2 specimens): HUMZ 95177, juvenile female, 584 mm TL, East China Sea (neurocranium, pectoral fin, pelvic fin and girdle); SU 13901, adult female, 730 mm TL, Japan (holotype of *Phaenopogon barbulifer*) (radiograph). *C. asper* (8 specimens): BMNH 1972.10.10.1 (holotype), adult male, 880 mm TL, Seychelles (radiograph and CT scan of head); SAIAB 6092, neonate male, 270 mm TL, unknown locality, South Africa (neurocranium); SAM 38269, adult female, 1045 mm TL, South Africa (all skeleton); UERJ 1641, adult male, 990 mm TL, Brazil (pelvic fin and girdle, claspers). UFPB 11862, juvenile male, 845 mm TL, Brazil (neurocranium, pectoral fin and girdle); UFPB 11863, juvenile female, 1076mm TL, Brazil (neurocranium, pectoral fin and girdle); UFPB 11864, juvenile female, 970mm TL (neurocranium); UFPB 11865, juvenile female, 1053mm TL, Brazil (neurocranium, pectoral fin and girdle). *C. australis* (5 specimens): AMS I 45670-001, juvenile male, 630 mm TL, Australia (radiograph); CSIRO H 5789-01 (holotype), adult female, 970 mm TL, Australia (radiograph); CSIRO H 7042-04, juvenile female, 605 mm TL, Australia (neurocranium, pectoral fin and girdle, pelvic fin and girdle); CSIRO H 7048-01, adult male, 993 mm TL, Australia (radiograph); NMNZ P 38074, adult male, 1020 mm TL, New Zealand (pelvic fin and girdle, claspers). *S. acanthias* (41 specimens): AMNH 38181, unknown sex and TL, Massachusetts, USA (all skeleton); AMNH 40802, neonate female, 190 mm TL, Massachusetts, USA (all skeleton); AMNH 97545, unknown sex and TL, USA (all skeleton); AMNH 97554, unknown sex and TL, USA (all skeleton); AMNH 97561, unknown sex and TL, USA (all skeleton); AMNH 53052, unknown sex and TL, USA (all skeleton); AMNH 225783, unknown sex and TL, off North Carolina, USA (all skeleton); BMNH 1888.2.6.72, adult male, 690 mm TL, United Kingdom (radiograph); BMNH 1929.10.20.1, adult female, 875 mm TL, United Kingdom (radiograph); BMNH 1931.8.10.1, adult male, 785 mm TL, New Zealand (syntype of *Squalus kirki*) (radiograph); BMNH 1976.7.30.20, adult female, 523 mm TL, France (radiograph); BMNH 1999.5.4.4, juvenile male, 550 mm TL, Falkland Islands, (neurocranium, pelvic fin and girdle, claspers); CAS 13381, adult male, 670 mm TL, Strait of Magellan, Chile (radiograph); HUMZ 30173, adult male, unknown TL, Argentina (neurocranium, pelvic fin and girdle, claspers); HUMZ 30291, adult male, 595 mm TL, Argentina, (pectoral fin and girdle, pelvic fin and girdle); HUMZ 107285, juvenile female, 340 mm TL, Argentina (neurocranium, pectoral fin); NMW 50118, adult male, 805 mm TL, Norway (all skeleton); NRM 85, neonate female, 177 mm TL, unknown locality, from the King Adolf Fredrik's collection at Ulriksdal (syntype of *S. acanthias*) (radiograph); NRM 21763, adult male, 645 mm TL, Öresund, near Råå, Sweden (radiograph); NRM 21757, adult male, 690 mm TL, Sweden (radiograph); NRM 21758, adult female, 715 mm TL, Sweden (radiograph); NRM 21759, adult female, 775 mm TL, Sweden (radiograph); NRM 21762, juvenile female, 420mm TL, Sweden (radiograph); NRM 21763, adult male, 645 mm TL, Sweden (radiograph); NRM 21767, adult male, 630 mm TL, Sweden (radiograph); NRM 44740, adult female, 830 mm TL, Sweden (radiograph); NRM 46694, unknown sex and TL, Atlantic Ocean (radiograph); NRM 46764, unknown sex and TL, Atlantic Ocean (radiograph); RMNH.PISC. 4315, juvenile female, 465 mm TL, The Netherlands (radiograph); RMNH.PISC. 27099, juvenile female, 466 mm TL, Schulpengat, The Netherlands (radiograph); SAIAB 21873, adult male, 675 mm TL, South Africa (neurocranium, pelvic fin and girdle, claspers); SAIAB 25918, adult male, 700 mm TL, South Africa (radiograph); SAIAB 26301, adult female, 670 mm TL, South Africa (radiograph); SAM 38271, juvenile male, 240 mm TL, unknown locality, Northwestern Atlantic Ocean; SAM 38276, adult male, 670 mm TL, unknown locality, Northwestern Atlantic Ocean (neurocranium, pectoral fin and girdle, pelvic fin and girdle, claspers); SAM 41629, adult female, 900 mm TL, unknown locality, Northwestern Atlantic Ocean (neurocranium); UUZM 159, juvenile male, 346 mm TL, unknown locality, from the King Adolf Fredrik's collection at Ulriksdal, Donation by J. Alströmer (syntype of *S. acanthias*) (radiograph); UUZM 160, neonate female, 380 mm TL, dried material, unknown locality, Donation by J. Alströmer (syntype of *S. acanthias*) (radiograph); UUZM 287, adult male, 700 mm TL, stuffed specimen, unknown locality, Donation by Gustav IV Adolf (syntype of *S. acanthias*) (radiograph); ZMH 101004, two juvenile males, 555, 635 mm TL, Canada (radiograph); ZMH 104416, adult male, 725 mm TL, Argentina (neurocranium, pelvic fin and girdle, claspers); ZMB 4504 adult male, 650 mm TL, The North Sea (pelvic fin and girdle). *S. suckleyi* (16 specimens): CAS 21424, neonate female, 285 mm TL, California, USA (all skeleton); CAS 21968, neonate female, 285 mm TL, California, USA (all skeleton); CAS 21971, neonate female, 290 mm TL, California, USA (all skeleton); CAS 34815, neonate male, 228 mm TL, California, USA (all skeleton); CAS 40592, neonate male, 185 mm TL, California, USA (all skeleton); CAS 40868, neonate female, 160 mm TL, California, USA (all skeleton); CAS 40873, neonate male, 330 mm TL, Washington State, USA (neurocranium, pelvic fin and girdle, claspers); CAS 56093, neonate male, 256 mm TL, California, USA (all skeleton); CAS 227267 (neotype of *S. suckleyi*), adult male, 674 mm TL, Washington, USA (pectoral girdle and fin, claspers) (radiograph); SAM 38346, adult female, 850 mm TL, California, USA (neurocranium, pectoral fin and girdle); HUMZ 87643, adult male, 665 mm TL, Japan (neurocranium, pelvic fin and girdle, claspers); HUMZ 87733, juvenile male, 495 mm TL, Japan (neurocranium); NSMT-P 79501, adult male, 740 mm TL, Japan (radiograph); NSMT-P 92640, adult female, 740 mm TL, Japan (radiograph); SAM 38355, unknown sex, 783 mm TL, California USA (neurocranium). *S. megalops* (1 specimen): AMS I 46093-001, adult male, 650 mm TL, Australia (neurocranium, pelvic fin and girdle, claspers). *S. brevirostris* (2 specimens): HUMZ 37664, adult female, unknown total length, Borneo (neurocranium, pelvic fin and girdle); HUMZ 189762, adult male, 433 mm TL, East China Sea (neurocranium, pelvic fin and girdle, claspers). *S. albifrons* (2 specimens): CSIRO H 2619-12, unknown sex and TL, Western Australia (neurocranium); MZUSP 121272 (formerly CSIRO uncatalogued), adult male, 760 mm TL, Australia (all skeleton). *S. mitsukurii* (2 specimens): HUMZ 68767, adult male, 620 mm TL, Emperor Sea Mount (neurocranium, pectoral fin and girdle); NSMT-P 44381, juvenile male, 770 mm TL, unknown locality, Southern Japan (neurocranium, pelvic fin and girdle, claspers). *S. montalbani* (1 specimen): MZUSP 121270 (formerly CSIRO uncatalogued), adult male, 713 mm TL, Australia (all skeleton). *S. japonicus* (3 specimens): HUMZ 80223, adult male, unknown TL, Japan (pectoral fin, pelvic fin and girdle, claspers); HUMZ 95213, unknown sex and TL, East China Sea (neurocranium); HUMZ 189737, adult male, 560 mm TL, East China Sea (neurocranium, pelvic fin and girdle, claspers). *D. licha* (2 specimens): SAIAB 6058, juvenile female, 406 mm TL, Mozambique (neurocranium, pectoral girdle and fin, pelvic girdle and fin). ZMA.PISC.112272, male, unknown locality (all skeleton from CT-scan data set provided in Brazeau et al., 2017a). *I. brasiliensis* (1 specimen): RMNH.PISC.37689, female, unknown locality (all skeleton from CT-scan data set provided in Brazeau et al., 2017b).
