# Supplementary material for: Untangling the systematic dilemma behind the roughskin spurdog Cirrhigaleus asper (Merrett, 1973) (Chondrichthyes: Squaliformes), with phylogeny of Squalidae and a key to Cirrhigaleus species
Source: PLoS One. 2023 Mar 6;18(3):e0282597. doi: 10.1371/journal.pone.0282597 (PMC9987817; doi:10.1371/journal.pone.0282597)
Supplement: Attachment — Submitted filename: Review cover letter FHM (2022-11-04).pdf [file pone.0282597.s011.pdf]

ELASMOBRANCH RESEARCH

Bonheiden, November 4th, 2022

Dear editors, authors,

It was my pleasure to read and review the paper by Viana & Soares entitled '*Untangling the systematic mystery behind the roughskin spurdog* Cirrhigaleus a*sper (Merrett, 1973) (Chondrichthyes: Squaliformes), with phylogeny of Squalidae and a key to species*'.

This topic is of taxonomic importance, and the paper itself is well organised, written and apparently prepared with much care as relatively little typo's and mistakes in cross-references to Figs etc. occur. It is also noticeable that authors are experts in this field.

Nevertheless, please allow me to suggest the following improvements (on headlines):

- **Titel**: avoid the word 'mystery' (perhaps better: dilemma), and be more specific about the key that is for the genus, whereas the title only mentions a single species and the family (now confusing).
- **Introduction**: visualise the main hypotheses by simplified trees in an additional Fig.
- **Morp. phyl. reconstruction**: there is a problem in the lettering of the Clades, either in the text of the MS, or in the Fig 13 itself (I guess in the latter). This needs special attention and if needed correction throughout the entire MS.
- **Key**: in terms of presentation, I think it would be better to place this key at the final end of the paper (i.e. after '*Taxonoic considerations within* Cirrhigaleus'), somewhat as a conclusion.
- **Figures**: perhaps better to put the lettering of all panels in CAPITALS (A, B, C…) for better contrast with abbreviations in Figs, and better contrast with Figs of external references. Also check for scale bars.
- **Smaller comments**: for several smaller comments (e.g., rows vs. files, holotype info *C. barbifer*, etc. etc.), please check the annotated MS carefully (see attached).

Wishing you all the best with finalising the MS,

Best regards,

Mollen.

Frederik Mollen
