# Supplementary material for: Untangling the systematic dilemma behind the roughskin spurdog Cirrhigaleus asper (Merrett, 1973) (Chondrichthyes: Squaliformes), with phylogeny of Squalidae and a key to Cirrhigaleus species
Source: PLoS One. 2023 Mar 6;18(3):e0282597. doi: 10.1371/journal.pone.0282597 (PMC9987817; doi:10.1371/journal.pone.0282597)
Supplement: Attachment — Submitted filename: Response to reviewers.docx [file pone.0282597.s013.docx]

**Response to Reviewers**

**By Viana and Soares**

**26 December 2022**

Dear Dr Kriwet,

Thank you for considering our manuscript for publication at PLOSOne and for your time spent and inputs provided during the review process.

We have addressed all the comments provided by you and the two reviewers. Answers to each comment and details about the changes made to the manuscript are provided below in blue.

**Journal requirements:**

1. Please ensure that your manuscript meets PLOS ONE's style requirements, including those for file naming.

This has been addressed accordingly.

2. Please state what role the funders took in the study. If the funders had no role, please state: "The funders had no role in study design, data collection and analysis, decision to publish, or preparation of the manuscript."

If this statement is not correct you must amend it as needed.

Please include this amended Role of Funder statement in your cover letter; we will change the online submission form on your behalf.

This has been addressed accordingly.

3. We note that you have referenced (ie. Bewick et al. [5]) which has currently not yet been accepted for publication. Please remove this from your References and amend this to state in the body of your manuscript: (ie “Bewick et al. [Unpublished]”) as detailed online in our guide for authors

This comment must be an editorial mistake as we did not reference to Bewick et al. [5] in this study. In our manuscript, reference [5] is as below:

[5] Fourmanoir P, Rivaton J. Poissons de la pente récifale externe de Nouvelle-Calédonie et des Nouvelles-Hébrides. Cahiers de l'Indo–Pacifique. 1979; 1(4):405–443.

Furthermore, we have not referenced any article that has not been accepted for publication. So, we have not removed or amended any of our references.

4. We note that Figure 1 in your submission contain [map/satellite] images which may be copyrighted.

We require you to either (1) present written permission from the copyright holder to publish these figures specifically under the CC BY 4.0 license, or (2) remove the figures from your submission:

Again, this comment must be an editorial mistake as Figure 1 does not correspond to (1) a map and (2) a figure with copyrighted image. So, we kept Figure 1 as it is in the revised version of this manuscript.

5. We note that Figure 25 in your submission contain copyrighted images.

We require you to either (1) present written permission from the copyright holder to publish these figures specifically under the CC BY 4.0 license, or (2) remove the figures from your submission:

a. Please upload the completed Content Permission Form or other proof of granted permissions as an "Other" file with your submission.

In the figure caption of the copyrighted figure, please include the following text: “Reprinted from [ref] under a CC BY license, with permission from [name of publisher], original copyright [original copyright year].”

We requested permission of the copyright holder and the Content Permission Form has been filled in and signed by both parties. The form has been uploaded to the system. Also, the figure caption has been amended as suggested.

**Additional Editor Comments**:

I agree with the second reviewer that it is unforunate that you did not provide the complete data matrix. I would suggest to include the full matrix in the manuscript and also a nexus file in the supplementary information, so that it is possible to recapitulate your analysis.

We included the summarized data matrix as supporting information (S1 and S2 Tables) in the original manuscript. Now, the full data matrix is provided as .txt and .xlxs files (S3 and S4 Files, respectively) containing the combined data matrix accordingly.

**Reviewers' comments:**

**Reviewer #1**:

Nevertheless, please allow me to suggest the following improvements (on headlines): -

Titel: avoid the word ‘mystery’ (perhaps better: dilemma), and be more specific about the key that is for the genus, whereas the title only mentions a single species and the family (now confusing).

We have replaced the word ‘mistery’ by ‘dilemma’ as suggested and added the genus-name to the title. Now the title is: ‘Untangling the systematic dilemma behind the roughskin spurdog *Cirrhigaleus* *asper* (Merrett, 1973) (Chondrichthyes: Squaliformes), with phylogeny of Squalidae and a key to *Cirrhigaleus* species’.

- Introduction: visualise the main hypotheses by simplified trees in an additional Fig.

We created a new figure for the revised version containing the main phylogenetic hypothesis. This figure is now placed in the Discussion section of the manuscript and it has been uploaded as Figure 14.

- Morp. phyl. reconstruction: there is a problem in the lettering of the Clades, either in the text of the MS, or in the Fig 13 itself (I guess in the latter). This needs special attention and if needed correction throughout the entire MS.

We have made a mistake when uploading the Figure 13 in the submission system. This is the reason why there is no correspondence between the clades in Figure and in text. We now have submitted the correct image for Figure 13 that contains the final phylogenetic tree of our analysis and the correct lettering for each clade. The text has been kept as in the original manuscript because it corresponds appropriately to what it is shown in the new Figure 13.

- Key: in terms of presentation, I think it would be better to place this key at the final end of the paper (i.e. after ‘Taxonoic considerations within Cirrhigaleus’), somewhat as a conclusion.

We have changed it accordingly in text. Now the identification key to *Cirrhigaleus* species is located after the subheading “Taxonomic considerations within Cirrhigaleus”.

- Figures: perhaps better to put the lettering of all panels in CAPITALS (A, B, C...) for better contrast with abbreviations in Figs, and better contrast with Figs of external references. Also check for scale bars.

We have changed all figures to Capital letters and checked all scale bars accordingly.

- Smaller comments: for several smaller comments (e.g., rows vs. files, holotype info C. barbifer, etc. etc.), please check the annotated MS carefully (see attached)

We have checked the full review file according for more specific comments.

**Reviewer #2:**

Overall a really good work with tons of information that can be put on larger context within Neoselachians. My only concern with the work is that the authors did not provide their full matrix, only the discrete features were provided. Consequently, there is no way evaluate their results. Furthermore, I would suggest to the authors to provide their matrices in nexus file to facilitate the revision of their coding, as their matrix seems to present some non-informative characters (these features do not provide grouping information, but can inflate the CI and RI of the tree).

We separated quantitative and qualitative characters used in the data matrix into two files of supporting information (S1 and S2 Tables) that were provided in the original manuscript. This information is present along the section ‘Material and Methods’ in which reads: “Character matrix was divided into two datasets: S1 Table, quantitative characters with absolute and normalized values for each terminal are presented; S2 Table, only qualitative characters are included.” As suggested, we have now uploaded the .txt and .xlxs files as a supporting information (S3 and S4 Files) since data was edited in Notepad++ and Microsoft Excel, respectively.

I have added some comments in a PDF of the paper (see comments with acrobat or other PDF readers, the browser one wont display them).

We have checked the full review file according for more specific comments.

**Comments to Review files**

**REVIEWER#1**

PAGE 11 LINE 254: `Based on the CI and RI provided are these two characters providing any grouping information’

Based only on CI and RI indexes, upper and lower teeth rows did not provide any grouping information in our analysis, but we prefer to keep these two characters since they vary among species and could be useful for future inferences.

PAGE 11 LINE 265: Separate both sets of characters, unless they where analysed together.

These characters were analyzed together so we are keeping the original text as it is.

PAGE 13 LINE 330: A similar structure was described by Maisey for hybodonts (see Maisey 1982, "The Anatomy and Interrelationships of Mesozoic Hybodont Sharks". The authors could provide differentiation between these structures to avoid confusion.

We appreciate the suggestion above. We have gone through Maisey (1982) that cited ‘caudal internasal keel’ (also cited as ‘median internasal keel’ in other studies such as Maisey 1983. cranial anatomy of H. basanus) in the ethmoidal region. The structure seems to be homologous to the subethmoidal ridge in Squaliformes as per Shirai’s (1992) definition but this is unclear. Rostral keel is an anterior-ventral extension of the subethmoidal ridge of Shirai. Because we are not describing the subethmoidal ridge in our study and because the scope of our study is on extant sharks and in-group relationships we prefer to do not mention this in text as it would require additional evidence (e.g. examination or review of fossil records, cranial definitions and studies). Furthermore, we would have to do the same for other skeletal structures. For these reasons we will not change the text here. However, we will consider this suggestion for an upcoming manuscript on the phylogeny of *Squalus*.

PAGE 17 LINE 400: Sensu Shirai 1992 and 1996. However, from what I have observed in Batomorphs this is not how the antorbital cartilages develop. Probably better to refer them as ecthemoid processes as they resemble more the structures present in Hexanchiformes. In the case of suggesting a homology between these structures and the ones on Batomorphs, I would ask the authors to provide ontogenetic evidence.

The objective of our study is not to provide homology between the anatomy of rays and sharks here. In order to replace the name of this structure we would have to review the structure’s name available in previous studies, suggest a definition and provide evidence but this is beyond the scope of our study. For these reasons and because we are following Shirai (1996) we will not make changes in the text here.

PAGE 17 LINE 402: sensu Shirai 1992 and 1996. However, these cartilages develop separately from the neurocranium in the Batomorphs I have studied (pers. observations). I would suggest changing the name of these structures in squalids to ecthedmoid processes. However, if the authors imply and homology between these "antorbital carilages" and the ones of batoids as the naming of the structure suggest, I would ask them to provide ontogenetic evidence to support this.

Same comment as above.

PAGE 17 LINE 408: Are characters 17 to 19 parsimony informative, if not please re-estimate the CI and RI values of the tree without them

Same comment as above.

PAGE 18 LINE 428: The basal condition for modern sharks and rays is the presence of three basal pectoral elements. The fusion of these elements in Dalatias and Isistius seems to be secondary. I would suggest the authors to change the coding of the states "0 to 1" and "1 to 0" just to indicate that the fusion is the derive feature or at least indicate this in the description of the feature, to avoid any confusions.

We have changed it accordingly (including the data matrix), excluding characters 17, 18 and 19 from the matrix which now contains 51 characters.

PAGE 31 LINE 764: Default value why not try more concavity values, see Goloboff et al., 2018 (<https://doi.org/10.1111/cla.12205>).

As suggested, we run different analyses testing three concavity values and the consensus tree obtained under k=1 was chosen as the preferred hypothesis.

PAGE 32 LINE 773: Remove non-informative characters and restimate scores

We removed non-informative characters 17, 18 and 19 and re-estimated scores as suggested.

**REVIEWER#2**

PAGE 1 LINE 1: Not a complete mystery, more a discussion. Perhaps better to use the word dilemma or something like this.

We have replaced the word ‘mistery’ by ‘dilemma’ as suggested and added the genus-name to the title. Now the title is: Untangling the systematic dilemma behind the roughskin spurdog *Cirrhigaleus* *asper* (Merrett, 1973) (Chondrichthyes: Squaliformes), with phylogeny of Squalidae and a key to *Cirrhigaleus* species

PAGE 1 LINE 3: Try to be more precise, bacause not clear for the (first) reader. In the title you mention in fact two taxon names: one species (C. asper) and one family (Squalidae) whereas the key is referred to the genus (not menionned).

See comment above.

PAGE 1 LINE 12: no ORCID?

Sarah has added her ORCID accordingly.

PAGE 1 LINE 14: E-mail:...

We have changed it accordingly.

PAGE 2 LINE 35: I suggest to be more precise as you describe both internal and external in the MS. So: clasper cartilages.

We have changed it as suggested.

PAGE 2 LINE 44: ? of conspicious cusplets

We have added the word ‘conspicuous’ as suggested.

PAGE 2 LINE 45: ? a

We have not made any changes here.

PAGE 3 LINE 55: Perhaps you can start to abbreviate genus name from here... to C. ?

We will keep providing full genus or species-name in the beginning of sentences. So, we have not made any changes here but if it applies to other parts of the manuscript we have done it so as suggested.

PAGE 3 LINE 72: Suggest to stress here that present (and not lack off...). Otherwise difficult to follow upon first reading.

We have replaced the word ‘lack’ by ‘absence’ before precaudal keel. Read it now as: “…along with absence of upper and lower precaudal pits, and absence of precaudal keels in *C. barbifer* are sufficient for…”.

PAGE 3 LINE 73: Please note that in your paper you notice such precaudal keel in the genus Squalus as well (character 48), so that it does not longer remain a proper character to seperate both genera. Did you stress this in your conclusions? Please check. I can't recall, and should read MS again.

Presence of precaudal keel has already been stressed as non-diagnostic in many previous studies (not ours!). We therefore think we have not made clear how previous studies have considered this character diagnostic or not. So, we have changed the text slightly here to clarify that. Please, read it now as: “For [18], [19] the nasal barbels … of upper and lower precaudal pits, and absence of precaudal keels in *C. barbifer* are sufficient for considering it as a valid and separate genus. Additional differences with *Squalus* …were noticed in [4] that supported its validity even though for these authors the lateral precaudal keel is also present in *Cirrhigaleus*.”

Moreover, we have added a statement under discussion (PAGE 39 LINE 957 of original manuscript) that this character is no longer diagnostic for *Cirrhigaleus* as previously proposed (not in our study). Please read it now as: “Similarities between Cirrhigaleus and Squalus regarding dentition, length of dorsal-fin spines, shape of dermal denticles, absence of nasal barbels, presence of precaudal keel… as well as some members of Squaliformes (e.g. *Centrophorus*).”

PAGE 4 LINE 78: ? C.

See related previous comment.

PAGE 5 LINE 105: Add '[23]' here? Now [23] is not mentionned in the text I think...

We have changed it accordingly. [23] is correctly assigned to the related reference in the References section.

PAGE 5 LINE 106: Suggestion: please consider to visualise the dilemma by adding a Figure that shows the two most important hypotheses in terms of simplified trees (see e.g. Fig. 52 in Villalobos-Segura et al. 2022 - Phylogeny Rays & Skates). <https://www.mdpi.com/1424-2818/14/6/456>

We have answered to this in previous comment of Reviewer#1.

PAGE 5 LINE 107: See comment on title.

We have replaced the word ‘mystery’ by ‘dilemma’ as suggested.

PAGE 5 LINE 116: Please allow me to suggest two additional references (i.e. pleas) here. ... following [Guinot et al. (2018), Mollen (2019)]. See: <https://www.mapress.com/zt/article/view/zootaxa.4461.1.8/0>

<https://www.mapress.com/zt/article/view/zootaxa.4571.2.13>

We appreciate the suggestions above but we think the protocols published in these studies do not apply here for what has been conducted in this manuscript. Moreover, methods of the teeth preparation and extraction has already been published elsewhere in several studies published by Viana (e.g. Viana et al. 2016) and we prefer to keep it consistent with that for comparative purposes.

What do you mean exactly?

We have clarified this in text. Please, now read it as: ‘…from upper and lower jaws (three lateral teeth from the first series of teeth, right side)…”

PAGE 6 LINE 137: Suggestion: stress/remind here that this is the type species. This will motivate your descision.

We have changed the text accordingly. Please read now as: “Descriptions are first given for the type-species, *C. barbifer,* followed by…”

PAGE 6 LINE 150: Be consistent in use of spaces. Se comment later.

We have checked it throughout the whole manuscript document.

PAGE 7 LINE 166: Add a comma here?

We have added a comma accordingly.

PAGE 9 LINE 206: Suggestion to update this 2016 reference in the reference list at the end. A new version was pubished by Sabaj in 2020 (Copeia).

We have updated it accordingly.

PAGE 9 LINE 217: Needed to add colour code here?

This was a mistake. We have excluded the color code here but kept it under the related figure caption. Please now read as: “mnl: medial nasal lobe supported by cartilage”

PAGE 10 LINE 245: it is

We have changed it accordingly. Now read as: “…whereas in *Cirrhigaleus* species it is 47–53.”

PAGE 11 LINE 254: See Cappetta 2012 (p. 9-10, fig. 1) for discussion on correct terminology: rows vs. files. In my opinion you need to use the word 'files' here (not 'rows').

We understand that the terminology of teeth is confusing and not yet completed revised and standardized. We follow Capetta (1987) who refers to file for teeth in the labio-lingual direction and row for mesio-distal direction. For these reasons, we have not changed the terminology in text.

PAGE 12 LINE 284: GENERAL COMMENT Author instructions ore not straight forward in this, but perhaps usefull to consider to change all lettering from panels in figures from small letters to CAPITAL LETTERS. Because small letters are also used in the Figures for anatomical abbreviations, it would make it easier to read the Figures. Capitals will make it also easier to differentiate figure references from this paper vs. figure references in other, referred papers.

We have added it accordingly.

PAGE 14 LINE 341: Add a dot.

We have added it accordingly.

PAGE 19 LINE 458: Add a comma.

We have added it accordingly.

PAGE 21 LINE 505: Attention: there is no letter 'c' in the actual Fig 8. You have 2x letter 'a' in the Fig. Please do not forget to correct the actual Fig. 8.

Should read 'c' I think.

We have corrected this accordingly in the figure caption and Figure 8.

PAGE 21 LINE 512: Add a dot.

We have added it accordingly.

PAGE 22 LINE 544: Forgot to align 'j' in Fig. 9 to the left? See my comment on the Figure itself.

We have corrected this accordingly in the figure caption and Figure 9.

PAGE 22 LINE 545: Small letter 'i'

We have corrected this accordingly in the figure caption and Figure 9.

PAGE 30 LINE 722: Elsewhere in the MS you also use the words 'cusplets'. If you mean the same, please be consistent throughout the MS. If different, I would suggest you to explain the difference in the MS.

Cusps and cusplets are different structures with different definitions. Cusplets are accessory structures usually smaller in size than the main cusp (in this case lateral cusp) and located medially to the main cusp. It is unnecessary to define cusp here as we are following the definition of cusp from Deynat and Seret (1996). We therefore clarified the term ‘cusplets’ under character 51 (page 30 line 736 of original manuscript). Please read as: “Cusplets are structures that are accessory to the main cusp and it is usually smaller in length. The cusplets are located medially to the lateral cusp on either side of the posterior margin of the crown base. In *C. asper* (Fig 12i–l) these cusplets are inconspicuous (51,0) as in *Squalus*. In *C. barbifer* and *C. australis* (Fig 12a–h) these cusplets are conspicuous (51,1).”

PAGE 30 LINE 727 and 728: To avoid confusion with body regions (samles taken from the flank, not dorsal), I would suggest to use the term 'occlusal' here in stead of dorsal.

Attention: the lettering 'a-e' and 'd-h' is overlapping, and needs correction. It is important to have the identification fo the panels right for the discussion that follows later.

We have changed the terminology here to ‘coronal view’ and corrected the figure lettering.

Please read as: “Fig 12. Scanning electron microscopy of dermal denticles of species of *Cirrhigaleus* in coronal (a–l, except h) and lateral (h) views.

*C. barbifer* (a–c): a: HUMZ 197852 (neotype), adult female, 870 mm TL; b: NMW 98257, adult female, 960 mm TL; c: CSIRO H 5875-09, adult female, 978 mm TL; *C. australis* (d–h): d: NMNZ P 38074, adult male, 1020 mm TL…”

PAGE 31 LINE 762: IMPORTANT COMMENT: the lettering of the Clades (A, B, C,...) does not conform with the lettering of the Clades (A, B, C,...) in Figure 13. Please check carefully throughout the entire MS. Perhaps you uploaded a wrong version of the Figure? Or you changed the lettering in the Figure, and forgot to change all in the MS? Please check carefully, it is important to have this right.

Please see comment below.

PAGE 32 LINE 795: ????? this is not the case in the actual Fig I guess you have uploaded the wrong version of the Fig...

We uploaded the wrong Figure 13 during the manuscript submission. Sorry! We now have uploaded the correct image and Figure 13 has the correct and corresponding clade lettering. The clade lettering in text is correct throughout the manuscript and no changes have been made in text.

PAGE 32 LINE 795: ????? this is not the case in the actual Fig. I guess you have uploaded the wrong version of the Fig...

Please see comment above.

PAGE 35 LINE 846: ? ... analysis, and may ...

We have corrected sentence accordingly. Please read now as: “Some synapomorphies, however, require revision as other members within Squaliformes that were left out of the present analysis.”

PAGE 35 LINE 849: ? ... [51], and the ...

We have corrected sentence accordingly. Please read now as: “Squalidae share the same conditions related to the number of pectoral basals with Etmopteridae and *Echinorhinus* [22], [37], [48], [67], and the number of pectoral articular regions with Chlamydoselachidae [32] and Etmopteridae [51]. ”

PAGE 37 LINE 901: Check lettering of clades, and suggest to be consistent in the use of a capital letter for the word 'Clade' throughout the MS.

We have corrected it accordingly throughout the manuscript.

PAGE 43 LINE 1049: In my opinion it would be more logical to put this title at the final end of the MS (i.e. after the title 'Taxonomic considerations within Cirrhigaleus'. Somewhat as a conclusion.

We have changed it accordingly in text. Now the identification key to *Cirrhigaleus* species is located after the subheading “Taxonomic considerations within *Cirrhigaleus*”.

PAGE 45 LINE 1095: I know, the holotype is lost (so you could only examine the published figure), but would it not make sense to mention the info (collection number, sex, location,...) of the holotype here? I am aware of the fact that you have all this info several pages later in the MS, but I think it would be of interest to mention it here in short.

We agree with the reviewer that the holotype information is relevant and must be provided. Please, be aware that we have already provided this in the original manuscript. It is located under the synonymy of *C. barbifer* and related authorship *(*PAGE 44 LINE 1070) as it is a standard practice in zoological taxonomy. So, for these reasons we have not changed location of this information in text.

PAGE 45 LINE 1112: Check your terminology on apron for consistency. Somethimes you use the word 'broad', somethimes 'wide'. Perhaps good to use the same throughout the MS.

We have changed it to “broad” throughout the manuscript as suggested. Also, to ‘narrow (instead of slender) whenever appropriate in the description of the apron.

PAGE 49 LINE 1168: See earlier comment on 'rows' vs. 'files'.

We understand that the terminology of teeth is confusing and not yet completed revised and standardized. We follow Capetta (1987) who refers to file for teeth in the labio-lingual direction and row for mesio-distal direction. For these reasons, we have not changed the terminology in text.

PAGE 50 LINE 1198: ? (a,b) ?'c,d'

We have corrected it accordingly. Please read now as: “Fig 17. First (a, b) and second (c, d) dorsal fins of *C. barbifer*, showing intraspecific regional variations.”

PAGE 52 LINE 1240: Please check carefully because there is an issue in the caption of the lettering of that specific Figure.

We have corrected it accordingly in text and Figure caption.

PAGE 53 LINE 1281: ? a

We have not made changes in text here.

PAGE 53 LINE 1347: OK to abbreviate New Caledonia in tables etc., but perhaps better to write in full in the text?

We have not made changes in text here as the name New Caledonia will be written several times in this section.

PAGE 64 LINE 1506: This seems to contradict your conclusion on 2112-2113, not?

In these lines you state that C. barbifer differs from C. asper (and australis) by a wide apron. But here you say that asper has a wide apron as well...

Sorry, our bad! We have corrected it accordingly in text. Please read now as: “…apron very short and narrow.”

PAGE 64 LINE 1507: See earlier comment.

We have addressed this in an earlier comment.

PAGE 67 LINE 1575: I think this is OK, but please double-check because there is a problem in the beginning of the caption/lettering in this Figure).

We have already addressed this throughout the manuscript.

PAGE 76 LINES 1780 AND 1792: Fig

We have corrected it accordingly in text. Please now read as: “Pectoral fins narrow (Fig 29a), although…”

PAGE 78 LINE 1822: Please check and correct - see earlier comments on caption/lettering of this Fig).

We have already addressed this throughout the manuscript.

PAGE 81 LINE 1887: Untill now you always put a space between the '%' and the 'CL' or 'TL' or ...I would make it consistent throughout the MS, especially in this chapter of the MS this is different.

We have corrected and standardized it accordingly throughout the text.

PAGE 83 LINE 1947: Use a dash in stead of hyphen.

We have corrected it accordingly throughout the text.

PAGE 90 LINES 2108 TO 2110: Do you mean the same in line 2106 and 2109-2110? I would suggest to be more consistent here.

We have already clarified in an earlier comment that the terms cusps and cusplets are distinct.

PAGE 90 LINE 2111: Not always, see your tooth formulas on lines 1167-1168. Seems to be variable within a single species as well. Would suggest to formulate a bit more 'safe'.

We have changed the text to correctly described that this character varies within a species. Please read now as: “Dental formula of *C. barbifer* is congruent with *C. asper* and *C. australis*, although some specimens of this species may present one intermediate tooth on upper and lower jaws (*vs*. absence of intermediate tooth).” Please, also note we have changed the word ‘median tooth’ to ‘intermediate tooth’ throughout the text for consistency.

PAGE 90 LINE 2112:? In line 1506 you state that apron is also markedly wide in C. asper...

We have addressed this in an earlier comment.

PAGE 93 LINE 2181: I think that more doi's can be given.

We have addressed this accordingly whenever possible.

PAGE 95 LINE 2214: Suggestion to insert/add two extra references here, i.e. Guinot et al. (2018) and Mollen (2019).

https://www.mapress.com/zt/article/view/zootaxa.4461.1.8/0

<https://www.mapress.com/zt/article/view/zootaxa.4571.2.13>

We have already answered to this suggestion in a previous comment. Please refer to it.

PAGE 97 LINE 2247: In fact, this is the name of the Series, with Matthias Stehmann as editor. Please correct reference accordingly.

We have corrected it accordingly.

PAGE 99 LINE 2331: I would suggest to update this reference, and refer to the published version of 2020 in Copeia. <https://doi.org/10.1643/ASIHCODONS2020>

We have updated this reference accordingly.

PAGE 100 LINE 2358: ? different style

We have addressed this accordingly.

FIGURE 15: Scale bars?

Unfortunately, we can not provide scale bars here as this picture was originally provided by one colleague who did not include a scale when took the pictures for referencing. For these reasons, we have not changed this image in the manuscript. In order to standardize this we have taken out the scale bars for all figures that provide full view of the specimens but we kept scales for close up images and illustrations whenever possible.

FIGURE 19: Scale bars?

Please see comment above.

FIGURE 21: Scale bars?

The original image does not have scale bars. So we have not changed this image.

FIGURE 25: Scale bars?

Please see comment above.

FIGURE 26: Scale bars?

Please see comment above.

FIGURE 29: Scale bars?

Please see comment above.
