# Supplementary material for: Untangling the systematic dilemma behind the roughskin spurdog Cirrhigaleus asper (Merrett, 1973) (Chondrichthyes: Squaliformes), with phylogeny of Squalidae and a key to Cirrhigaleus species
Source: PLoS One. 2023 Mar 6;18(3):e0282597. doi: 10.1371/journal.pone.0282597 (PMC9987817; doi:10.1371/journal.pone.0282597)
Supplement: Attachment — Submitted filename: Response to Reviewers.docx [file pone.0282597.s014.docx]

**Response to Reviewers**

**By Viana and Soares**

**9 February 2023**

Dear Dr Kriwet,

Thank you for considering our manuscript for publication at PLOSOne and for your time spent and inputs provided during the review process.

We have addressed all the comments provided by you and the two reviewers. Answers to each comment and details about the changes made to the manuscript are provided below in blue.

**Journal Requirements:**
Please review your reference list to ensure that it is complete and correct. If you have cited papers that have been retracted, please include the rationale for doing so in the manuscript text, or remove these references and replace them with relevant current references. Any changes to the reference list should be mentioned in the rebuttal letter that accompanies your revised manuscript. If you need to cite a retracted article, indicate the article’s retracted status in the References list and also include a citation and full reference for the retraction notice.
We have reviewed accordingly and we have not made any changes. We did not cite any paper that has been retracted.

**Additional Editor Comments:**

Dear authors:
Thank you very much for considerng the comments by the reviewers and also for your patience. Reviewer 2 has raised an important aspect concerning character 16. Please check this comment and address it shortly in your manuscript. Please also check Figures 14 and 15 as there quality seems to be very low and improve the quality if necessary.
Please re-submit the manuscript once you have done the required improvements. I will check it (without another reviewer round) and make asap a final decision.
Kind regards,
Jürgen

We have addressed the issues raised with character 16 and the quality of the Figures 14 and 15.
 **Reviewers' comments:

Reviewer's Responses to Questions**

**Comments to the Author**

1. If the authors have adequately addressed your comments raised in a previous round of review and you feel that this manuscript is now acceptable for publication, you may indicate that here to bypass the “Comments to the Author” section, enter your conflict of interest statement in the “Confidential to Editor” section, and submit your "Accept" recommendation.

Reviewer #1: All comments have been addressed

Reviewer #2: All comments have been addressed

2. Is the manuscript technically sound, and do the data support the conclusions?

The manuscript must describe a technically sound piece of scientific research with data that supports the conclusions. Experiments must have been conducted rigorously, with appropriate controls, replication, and sample sizes. The conclusions must be drawn appropriately based on the data presented.

Reviewer #1: (No Response)

Reviewer #2: Yes

3. Has the statistical analysis been performed appropriately and rigorously?

Reviewer #1: (No Response)

Reviewer #2: N/A

4. Have the authors made all data underlying the findings in their manuscript fully available?

The [PLOS Data policy](http://www.plosone.org/static/policies.action#sharing) requires authors to make all data underlying the findings described in their manuscript fully available without restriction, with rare exception (please refer to the Data Availability Statement in the manuscript PDF file). The data should be provided as part of the manuscript or its supporting information, or deposited to a public repository. For example, in addition to summary statistics, the data points behind means, medians and variance measures should be available. If there are restrictions on publicly sharing data—e.g. participant privacy or use of data from a third party—those must be specified.

Reviewer #1: (No Response)

Reviewer #2: (No Response)

5. Is the manuscript presented in an intelligible fashion and written in standard English?

PLOS ONE does not copyedit accepted manuscripts, so the language in submitted articles must be clear, correct, and unambiguous. Any typographical or grammatical errors should be corrected at revision, so please note any specific errors here.

Reviewer #1: (No Response)

Reviewer #2: (No Response)

6. Review Comments to the Author

Please use the space provided to explain your answers to the questions above. You may also include additional comments for the author, including concerns about dual publication, research ethics, or publication ethics. (Please upload your review as an attachment if it exceeds 20,000 characters)

Reviewer #1: (No Response)

Reviewer #2: Char. 16. While I agree, the objective of the present work is not to address the homology of structures between batoids and sharks or in group shark relations. By naming a character, for example, Antorbital cartilages in the neurocranium, some sense of homology is implied in the name, the limitations on the generalisations on the observations need to be adequately clarified in their character description, so the reader interprets no untested homology. While I do not expect a full anatomical revision, I would hope that the authors include a short statement mentioning that while they are using the term Antorbital cartilage, no homology between this observed feature and the one present on batoids is implied by them as this need to be further tested. The same goes for characters 9 and 10. Furthermore, if they are following Shirai (1992 and 1996) in the codification of character 16 for this paper need to be cited to add context to their character description, same goes for Maisey's works (1982, 1983).

We have changed the text accordingly to clarify description of Character 16. Please read as: “16) Antorbital cartilage…. No homology between this character and the one similarly described for non-squalean sharks and batoids in Shirai (1992) is implied in the present study as this requires further investigation. Character codification follows Shirai (1992, 1996).”

However, again we did not do the same for character 9 and 10 (rostral keel) as the ‘caudal internasal keel’ (also cited as ‘median internasal keel’ in other studies such as Maisey 1982, 1983) seems to be homologous to the subethmoidal ridge in Squaliformes as per Shirai’s (1992) and the present study. The character rostral keel does not correspond to this structure. As we clarified in the first round of the review process, the rostral keel is an anterior-ventral extension of the subethmoidal ridge..

7. PLOS authors have the option to publish the peer review history of their article ([what does this mean?](https://journals.plos.org/plosone/s/editorial-and-peer-review-process#loc-peer-review-history)). If published, this will include your full peer review and any attached files.


If you choose “no”, your identity will remain anonymous but your review may still be made public.


**Do you want your identity to be public for this peer review?** For information about this choice, including consent withdrawal, please see our [Privacy Policy](https://www.plos.org/privacy-policy).

Reviewer #1: **Yes:** Frederik H. Mollen (Elasmobranch Research, Belgium)

Reviewer #2: **Yes:** Eduardo Villalobos Segura
